# Diagnostic Insights Into Pathogen Spectrum and Mixed Microbial Detection in Critically Ill Patients With Pulmonary Infection Using Targeted Next‐Generation Sequencing

**DOI:** 10.1155/carj/8288339

**Published:** 2026-07-24

**Authors:** Hui Zhang, Lin Yang, Zhao Zhao, Li Zhao, Shujuan Zhang, Xiaozhi Jiang, Fengdan Liu, Jin Wu, Lili Du, Guannan Ma, Peili Chen

**Affiliations:** ^1^ Department of Critical Care Medicine, Shangqiu First People’s Hospital, Shangqiu, Henan, China; ^2^ Engineering Research Center for Sepsis Diagnosis and Treatment of Henan Province, Shangqiu, Henan, China; ^3^ Zhejiang Key Laboratory of Digital Technology in Medical Diagnostics, Hangzhou, Zhejiang, China; ^4^ Chongqing Precision Medicine Industry Technology Research Institute, Chongqing, China

**Keywords:** critically ill patients, diagnostic performance, mixed microbial infection, pulmonary infection, targeted next-generation sequencing (tNGS)

## Abstract

**Background:**

Pulmonary infections are common and potentially life‐threatening in critically ill patients. Conventional microbiological tests (CMTs) often show limited sensitivity, particularly in the setting of prior antimicrobial exposure. Targeted next‐generation sequencing (tNGS) has emerged as an alternative approach for broad pathogen detection; however, data describing pathogen spectrum, mixed infection patterns, and diagnostic performance in critically ill populations remain limited.

**Methods:**

This retrospective study included 217 critically ill patients with suspected pulmonary infection who underwent tNGS testing of respiratory specimens. Baseline clinical characteristics were summarized. The pathogen spectrum and mixed detection patterns identified by tNGS were analyzed, including mixed microbial codetection. Diagnostic performance of tNGS was compared with that of CMTs using clinical diagnosis as the reference standard. Sensitivity, specificity, accuracy, agreement indices, and McNemar’s test were applied.

**Results:**

tNGS detected at least one microorganism in 208 of 217 patients, yielding a significantly higher overall detection rate than CMTs. A broad spectrum of bacterial, viral, and fungal pathogens was identified, and mixed detections were common, frequently involving organisms from different pathogen categories. Co‐occurrence network analysis highlighted recurrent patterns of mixed microbial detection in this cohort. Compared with CMTs, tNGS demonstrated substantially higher sensitivity and overall diagnostic accuracy, whereas conventional methods showed higher specificity.

**Conclusions:**

tNGS provides a comprehensive overview of the pathogen spectrum and mixed detection patterns in critically ill patients with suspected pulmonary infection. Compared with CMTs, tNGS offers a markedly higher detection rate and sensitivity. However, careful clinical interpretation remains essential, particularly in the context of frequent mixed microbial detections, to ensure appropriate integration of sequencing results into clinical decision‐making.

## 1. Introduction

Pulmonary infections are among the most common causes of morbidity and mortality in critically ill patients, particularly in the intensive care unit (ICU) [1–3]. Severe pneumonia and secondary pulmonary infections frequently complicate the clinical course of critically ill patients and are associated with prolonged mechanical ventilation, increased length of hospital stay, and increased mortality [4]. Early identification of causative pathogens is therefore essential for the timely initiation of appropriate antimicrobial therapy and improvement of clinical outcomes.

However, the diagnosis of pulmonary infections in critically ill patients remains challenging. Clinical manifestations are often nonspecific, and radiological findings may overlap with noninfectious conditions, such as pulmonary edema or acute respiratory distress syndrome (ARDS). In addition, prior exposure to broad‐spectrum antimicrobial agents is common in this population, which substantially reduces the diagnostic yield of traditional microbiological methods [5, 6].

Conventional microbiological tests (CMTs), including culture‐based methods, antigen detection, and targeted polymerase chain reaction assays, remain the cornerstone of pathogen identification in clinical practice. Nevertheless, these approaches have several well‐recognized limitations [7, 8]. Culture‐based methods are time‐consuming and show reduced sensitivity following antimicrobial exposure, while targeted molecular assays are limited to predefined pathogens and fail to detect unexpected or rare microorganisms [9–11].

Previous studies have demonstrated that the overall pathogen detection rate of CMTs in patients with pneumonia, particularly in critically ill populations, is suboptimal [12]. Consequently, a substantial proportion of patients receive empirical antimicrobial therapy without microbiological confirmation, which may contribute to inappropriate antimicrobial use and the development of antimicrobial resistance [13].

Next‐generation sequencing–based approaches have emerged as promising diagnostic tools for infectious diseases [14]. By enabling simultaneous detection of a broad range of bacterial, viral, and fungal pathogens in a single assay, targeted or metagenomic next‐generation sequencing provides a culture‐independent method for pathogen identification [15, 16]. Several studies have shown that next‐generation sequencing can improve pathogen detection compared with CMTs, particularly in cases of severe, atypical, or culture‐negative infections [17].

In respiratory infections, targeted next‐generation sequencing (tNGS) has been increasingly applied to bronchoalveolar lavage fluid (BALF) and other respiratory specimens, demonstrating broader pathogen coverage and higher sensitivity [18, 19]. However, most existing studies have primarily focused on diagnostic yield or sensitivity [20]. Data regarding pathogen spectrum revealed by next‐generation sequencing in critically ill patients remain limited, especially in real‐world ICU settings.

Therefore, the present study aimed to characterize the pathogen spectrum and mixed microbial patterns detected by tNGS in critically ill patients with pulmonary infections and to compare its diagnostic performance with that of CMTs.

## 2. Methods

### 2.1. Study Design and Patient Population

This retrospective observational study was conducted in critically ill patients admitted to the ICU of the First People’s Hospital of Shangqiu. Consecutive adult patients with suspected pulmonary infections who underwent tNGS testing of respiratory specimens between December 2022 and April 2024 were screened for eligibility.

Patients were eligible for inclusion if they met the following criteria: clinical suspicion of pulmonary infection based on compatible symptoms (e.g., fever, cough, and dyspnea), laboratory abnormalities, and new or progressive radiological findings. Patients without a clear clinical diagnosis of pulmonary infection after comprehensive evaluation or with incomplete clinical data were excluded. The final study cohort consisted of 217 patients.

### 2.2. Clinical Data Collection

Demographic characteristics (age and sex), underlying comorbidities, primary diagnosis at ICU admission, and organ dysfunction or complications during hospitalization were extracted from the electronic medical record system. Organ dysfunction was defined according to established clinical criteria and included respiratory failure, ARDS, sepsis or septic shock, acute kidney injury, and liver dysfunction.

The study cohort included 217 critically ill patients with suspected pulmonary infection, categorized into general pulmonary infection (*n* = 112), severe pneumonia (*n* = 83), and aspiration‐related pneumonia (*n* = 22). The final clinical diagnosis was established by attending ICU physicians based on integrated assessment of clinical manifestations, radiological findings, microbiological results, and response to antimicrobial therapy.

### 2.3. Sample Collection and CMTs

Respiratory specimens, including BALF, sputum, or other respiratory tract samples, were collected according to standardized clinical procedures. BALF was preferentially obtained when bronchoscopy was clinically feasible. Specimens were typically collected within 72 h of ICU admission (1–5 days). Due to the retrospective design and frequent altered mental status or prior intubation in critically ill patients, the exact date of symptom onset was inconsistently documented and could not be reliably determined for all patients.

CMTs included bacterial and fungal cultures, acid‐fast staining and culture for Mycobacterium tuberculosis, and targeted molecular or serological assays (e.g., PCR or antigen testing) when clinically indicated. All conventional microbiological results were recorded and compared with the results of tNGS.

Specimens were processed for tNGS according to standard laboratory protocols. The timing of specimen collection reflects routine clinical practice, where sampling was performed pragmatically rather than at standardized research time points.

### 2.4. tNGS

tNGS was performed on respiratory specimens in accordance with the manufacturer’s instructions. Briefly, total nucleic acids were extracted from clinical samples, followed by reverse transcription (for RNA viruses), library construction, and sequencing using a targeted respiratory pathogen panel. The panel was designed to detect common bacterial, viral, fungal, Mycoplasma, and Chlamydia pathogens associated with pulmonary infections through ultra‐multiplex PCR amplification. Sequencing was performed on the Oxford nanopore platform. The assay utilized a targeted respiratory pathogen panel encompassing approximately 354 bacterial, viral, and fungal pathogens.

Sequencing data were analyzed using a standardized bioinformatics pipeline provided by the sequencing company. Low‐quality reads and adapter sequences were removed, and the remaining reads were aligned to a curated clinical pathogen database. Pathogen identification was based on predefined criteria. A microorganism was considered positive if the number of pathogen‐specific reads exceeded the established reporting threshold, taking into account background reads in negative controls. For pathogens detected in negative controls, a reads‐per‐million (RPM) ratio ≥ 10 was considered positive. For pathogens without background reads in negative controls, an RPM ≥ 0.05 was used as the cutoff for positive detection. While tNGS results were available to attending ICU physicians, antimicrobial therapy decisions were made at clinical discretion.

### 2.5. tNGS Positivity Criteria and Quantitative Indicators

Pathogen‐specific normalized read counts were used to define tNGS positivity. For bacteria, fungi, atypical pathogens, and viruses, a normalized read count ≥ 10 was required; for Mycobacterium tuberculosis complex, a normalized read count ≥ 1 was required. These thresholds help distinguish probable pathogens from low‐level background signals. Clinical relevance of each pathogen was further adjudicated based on clinical manifestations, conventional microbiology, and treatment response. Mixed microbial detection was defined as the presence of two or more pathogens meeting these criteria in the same respiratory specimen.

### 2.6. Diagnostic Performance Evaluation

The diagnostic performance of tNGS and CMTs was evaluated using the clinical diagnosis as the reference standard. True‐positive, false‐positive, false‐negative, and true‐negative results were determined for each diagnostic method. Sensitivity, specificity, positive predictive value (PPV), negative predictive value (NPV), accuracy, Youden index, balanced accuracy, F1 score, positive likelihood ratio, negative likelihood ratio, and Cohen’s kappa coefficient were calculated to assess diagnostic performance and agreement.

### 2.7. Statistical Analysis

Categorical variables were expressed as numbers and percentages, and continuous variables were presented as mean ± standard deviation or median (interquartile range), as appropriate. Diagnostic performance metrics were calculated with corresponding 95% confidence intervals. McNemar’s test was used to compare paired proportions between tNGS and CMTs. A two‐sided *p* value < 0.05 was considered statistically significant. Statistical analyses were performed using *R* Version 4.2.2. Network visualization and figure generation were performed using the *R* software.

### 2.8. Ethical Approval

This study was approved by the Institutional Ethics Committee of the First People’s Hospital of Shangqiu (HS2025092). Given the retrospective nature of the study, the requirement for informed consent was waived.

## 3. Result

### 3.1. Baseline Clinical Characteristics

A total of 217 critically ill patients with suspected pulmonary infection were enrolled in this study. The baseline clinical characteristics are summarized in Table 1. Among these patients, 158 were male (72.81%) and 59 were female (27.19%), with a mean age of 63 ± 16.6 years. Patients aged 60–79 years constituted the largest age group (109/217, 50.23%), followed by those aged 40–59 years (46/217, 21.20%), ≥ 80 years (33/217, 15.21%), and < 40 years (29/217, 13.36%).

**TABLE 1:** Baseline clinical characteristics of critically ill patients undergoing pulmonary tNGS testing.

Characteristic	*n*	Percentage (%)
Gender	217	
Male	158	72.81
Female	59	27.19
Age, years		
Mean ± SD	63 ± 16.6	
40–59	46	21.20
60–79	109	50.23
≥ 80	33	15.21
Comorbidities		
Diabetes mellitus	52	23.96
Cardiovascular or cerebrovascular disease	66	30.41
Chronic lung disease (COPD, bronchiectasis, ILD, etc.)	19	8.76
Malignancy	10	4.61
Chronic kidney disease/renal insufficiency	7	3.23
Primary Diagnosis on Current Admission		
Neurological critical illness or trauma	152	70.05
Pulmonary infection	112	51.61
Severe pneumonia	83	38.25
Aspiration‐related pneumonia	22	10.14
Abdominal infection/peritonitis	18	8.29
Organ Dysfunction and Complications		
Heart failure	40	18.43
Renal insufficiency	46	21.20
Hepatic dysfunction	57	26.27
Gastrointestinal complications	50	23.04
Thrombosis	29	13.36
Hypoproteinemia/nutritional risk	173	79.72
Electrolyte and acid‐base disorders	21	9.68

Pre‐existing comorbidities were frequently observed. Hypertension was the most common comorbidity (105/217, 48.39%), followed by diabetes mellitus (52/217, 23.96%), cardiovascular or cerebrovascular disease (66/217, 30.41%), chronic lung disease, including COPD, bronchiectasis, and interstitial lung disease (19/217, 8.76%), malignancy (10/217, 4.61%), and chronic kidney disease or renal insufficiency (7/217, 3.23%). Aspiration‐related pneumonia was present in 22 patients (10.14%) and abdominal infection/peritonitis in 18 patients (8.29%).

Regarding primary diagnoses on admission, the three most common categories were neurological critical illness or trauma (152/217, 70.05%), pulmonary infection (112/217, 51.61%), and severe pneumonia (83/217, 38.25%). Organ dysfunction and complications during hospitalization were frequently observed, including respiratory failure (103/217, 47.47%), hepatic dysfunction (57/217, 26.27%), renal insufficiency (46/217, 21.20%), heart failure (40/217, 18.43%), gastrointestinal complications (50/217, 23.04%), thrombosis (29/217, 13.36%), hypoproteinemia/nutritional risk (173/217, 79.72%), and electrolyte or acid‐base disorders (21/217, 9.68%). No statistically significant differences in polymicrobial detection were observed among pneumonia subtypes, including pulmonary infection, severe pneumonia, and aspiration‐related pneumonia (*p* = 0.844), although bacterial detection was more frequent in aspiration‐related pneumonia (Table S1). Collectively, these findings indicate that the cohort reflected a clinically heterogeneous ICU population with suspected pulmonary infections encountered in real‐world critical care practice.

### 3.2. Pathogen Spectrum Detected by tNGS

**FIGURE 1:**
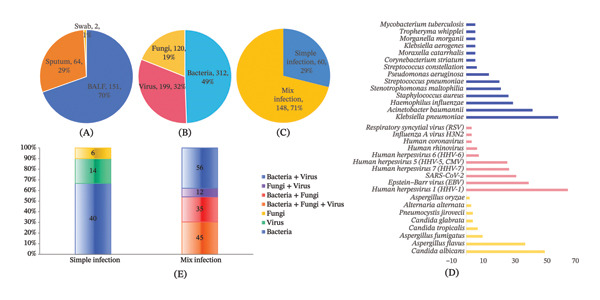
Pathogen spectrum and mixed infection patterns detected by targeted next‐generation sequencing (tNGS) in critically ill patients with suspected pulmonary infections. (A) Distribution of specimen types submitted for tNGS analysis, including bronchoalveolar lavage fluid (BALF), sputum, and swab samples. (B) Composition of detected pathogens classified as bacteria, viruses, and fungi. (C) Proportion of simple infections and mixed infections among all tNGS‐positive cases. (D) Frequency distribution of the most common bacterial, viral, and fungal pathogens identified by tNGS. (E) Comparison of pathogen categories between simple infection and mixed infection groups.

tNGS detected at least one microorganism in 208 of the 217 patients. The distribution of specimen types is shown in Figure 1A, with BALF accounting for the majority of samples (151/217, 70%), followed by sputum (64/217, 29%) and swab specimens (2/217, 1%).

Across all detected microorganisms, bacteria constituted the largest proportion (49.4%), followed by viruses (31.5%) and fungi (19%) (Figure 1B). Among the 208 tNGS‐positive patients, both simple and mixed microbial detections were identified (Figure 1C). Among the 208 tNGS‐positive patients, single‐microorganism detection was observed in 60 patients (29%), whereas mixed microbial detection, defined as ≥ 2 microorganisms meeting predefined tNGS positivity criteria in the same specimen, was observed in 148 patients (71%). These findings reflect sequencing‐level codetections and do not automatically indicate clinically confirmed coinfections.

Within the simple infection group, bacterial‐only detections were most frequent (40/60, 66.7%), followed by virus‐only (14/60, 23.3%) and fungi‐only detections (6/60, 10.0%) (Figure 1D). In patients with mixed microbial detections, codetection across different pathogen categories was common (Figure 1E), including bacteria plus virus (56/148, 37.8%), bacteria plus fungi (35/148, 23.6%), bacteria plus fungi plus virus (45/148, 30.4%), and fungi plus virus (12/148, 8.1%).

Stratified analyses were performed to examine pathogen detection patterns across key clinical variables (Table S1). tNGS positivity was consistently high across all subgroups, ranging from 95.4% to 97% across age groups. Polymicrobial detection was more frequent in patients aged ≥ 80 years (72.7%) compared with those aged 40–59 years (65.2%; *p* = 0.772) and was higher in BALF specimens than in sputum (70% vs. 62%). Most comorbidities and ICU diagnostic categories had limited impact on polymicrobial detection.

### 3.3. Pathogen Co‐Occurrence Network Revealed by tNGS

**FIGURE 2:**
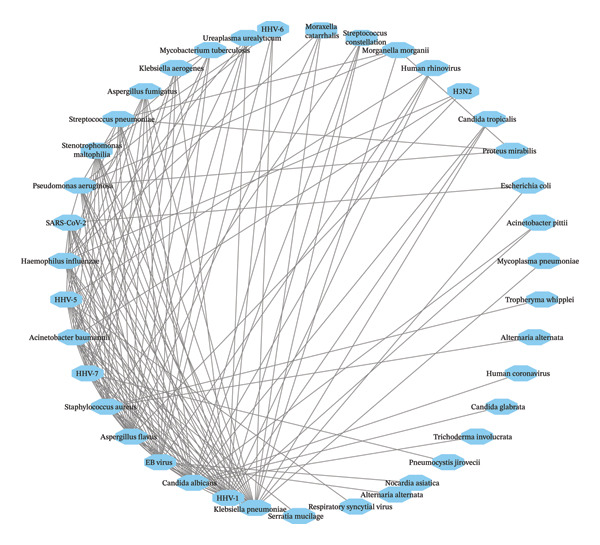
Co‐occurrence network of pulmonary pathogens detected by targeted next‐generation sequencing (tNGS). Each node represents a pathogen identified by tNGS, and edges indicate codetection of two pathogens within the same clinical sample. The network illustrates complex mixed microbial infection patterns in critically ill patients, highlighting frequent coinfections involving bacterial, viral, and fungal pathogens.

To characterize mixed microbial detection patterns, a pathogen co‐occurrence network was constructed based on tNGS results (Figure 2). In this network, each node represents a detected microorganism, and edges indicate codetection of two microorganisms within the same respiratory specimen.

As shown in Figure 2, the network displayed a dense connectivity pattern, reflecting frequent codetection of multiple microorganisms in this ICU population. Co‐occurrence was observed both within and across pathogen categories, with bacterial–viral, bacterial–fungal, and viral–fungal associations widely distributed. Several microorganisms occupied central positions in the network, including common ICU‐associated bacteria, such as *Klebsiella pneumoniae, Acinetobacter baumannii, Pseudomonas aeruginosa, Stenotrophomonas maltophilia, Staphylococcus aureus, Haemophilus influenzae*, and *Streptococcus pneumoniae*. Frequently detected viruses, including *HHV-1, HHV-5, HHV-6, HHV-7*, *Epstein–Barr virus,* and *SARS-CoV-2*, as well as fungi, such as *Candida albicans*, *Aspergillus flavus*, and *Aspergillus fumigatus*, were also prominently represented. In contrast, organisms with fewer connections, such as *Mycoplasma pneumoniae*, *Pneumocystis jirovecii*, and *Nocardia asiatica*, were located at the network periphery, indicating more sporadic codetection patterns.

### 3.4. Diagnostic Performance of tNGS

**FIGURE 3:**
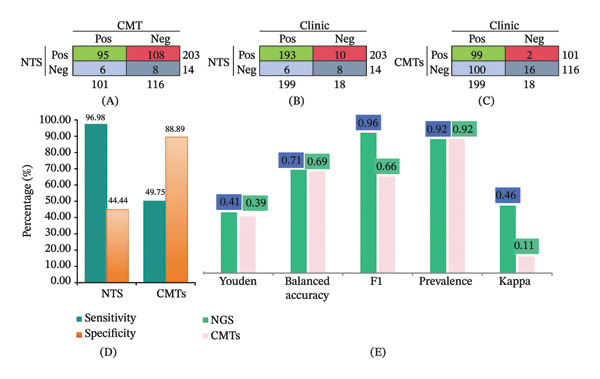
Diagnostic performance comparison of targeted next‐generation sequencing (tNGS) and conventional microbiological tests (CMTs) using clinical diagnosis as the reference standard. (A–C) Confusion matrices illustrating the diagnostic classification of tNGS and CMTs. (A) Cross‐comparison between tNGS and CMTs. (B) Comparison of tNGS results with clinical diagnosis. (C) Comparison of CMT results with clinical diagnosis. The numbers in each matrix represent true‐positive (TP), false‐positive (FP), false‐negative (FN), and true‐negative (TN) results. (D) Comparison of sensitivity and specificity between tNGS and CMTs using clinical diagnosis as the reference standard. (E) Comparison of overall diagnostic performance between tNGS and CMTs based on Youden index, balanced accuracy, F1 score, positive detection prevalence, and Cohen’s kappa coefficient.

Using clinical diagnosis as the reference standard, the diagnostic performance of tNGS and CMTs was evaluated (Table 2, Figure 3). tNGS showed a markedly higher sensitivity than CMTs, with sensitivities of 97.0% and 49.7%, respectively. The overall diagnostic accuracy of tNGS was also higher than that of CMTs. In contrast, CMTs demonstrated a higher specificity than tNGS.

**TABLE 2:** Diagnostic performance of targeted next‐generation sequencing and conventional microbiological tests.

Metric	NGS	CMTs
TP	193	99
FP	10	2
FN	6	100
TN	8	16
N	217	217
Sensitivity	97.0% (93.6%–98.9%)	49.7% (42.6%–56.9%)
Specificity	44.4% (21.5%–69.2%)	88.9% (65.3%–98.6%)
PPV	95.1% (91.1%–97.6%)	98.0% (93.0%–99.8%)
NPV	57.1% (28.9%–82.3%)	13.8% (8.1%–21.4%)
Accuracy	92.6% (88.3%–95.7%)	53.0% (46.1%–59.8%)
Youden	41.40%	38.60%
Balanced accuracy	70.70%	69.30%
F1	0.96	0.66
Prevalence	0.917	0.917
LR+ (HA)	1.751 (1.157–2.648)	3.781 (1.016–14.071)
LR–(HA)	0.073 (0.028–0.186)	0.579 (0.467–0.717)
Kappa	0.461	0.111

tNGS identified 193 true‐positive cases, whereas CMTs detected 99 true‐positive cases, corresponding to a substantially lower false‐negative rate for tNGS. Composite performance metrics further distinguished the two methods. The F1 score was 0.96 for tNGS and 0.66 for CMTs. The Youden index was 0.41 for tNGS and 0.39 for CMTs, and the balanced accuracy was 70.7% and 69.3%, respectively. Agreement with clinical diagnosis, assessed using Cohen’s kappa coefficient, was moderate for tNGS (*κ* = 0.461) and slight for CMTs (*κ* = 0.111). The positive percent agreement was higher for tNGS than for CMTs (97.0% vs. 49.7%), whereas the negative percent agreement was lower (44.4% vs. 88.9%). The corresponding confusion matrices are presented in Figure 3.

The positive detection rates of the two methods were further compared using McNemar’s test (Table 3). The positive rate of tNGS was significantly higher than that of CMTs (95.9% vs. 46.5%, *p* < 0.001). A significant difference in sensitivity between the two methods was also observed (*p* < 0.001), whereas CMTs showed a significantly higher specificity than tNGS (*p* = 0.013).

**TABLE 3:** Comparison of pathogen detection rates between targeted next‐generation sequencing and conventional microbiological tests.

Detection method	tNGS (*n* = 217)	CMTs (*n* = 217)	McNemar
Positive	208 (95.9%)	101 (46.5%)	1.7068E‐15
Negative	9 (4.1%)	116 (53.4%)	

Sensitivity	96.98%	49.75%	1.6709E‐19

Specificity	44.44%	88.89%	0.01332833

## 4. Discussion

In this cohort of 217 critically ill patients with suspected pulmonary infection, the study population was characterized by advanced age, a high burden of comorbidities, and frequent organ dysfunction, reflecting the clinical complexity commonly encountered in the ICU setting. In such patients, timely identification of etiologic pathogens is particularly challenging, as empirical broad‐spectrum antimicrobial therapy is often initiated before microbiological results are available. Current guidelines therefore emphasize early acquisition of appropriate respiratory specimens to support subsequent diagnostic refinement and antimicrobial optimization once microbiological evidence becomes available [21, 22].

The diagnosis of pulmonary infections in critically ill patients remains challenging. Clinical manifestations are nonspecific, and radiological findings may overlap with noninfectious conditions. Prior exposure to broad‐spectrum antimicrobial agents, often initiated empirically for severe pneumonia, sepsis, or septic shock, can further reduce the yield of conventional culture‐based methods [23]. ICU respiratory infections are also increasingly complicated by ventilator‐associated pneumonia, multidrug‐resistant organisms, and biofilm‐associated pathogens. Active nasal swab surveillance has been proposed to identify high‐risk bacteria and prevent VAP [24]. Chronic *P. aeruginosa* pulmonary biofilm infection is difficult to diagnose, and tesG expression may serve as a potential biomarker [25]. *A. baumannii*–associated pneumonia represents another major therapeutic challenge due to increasing antimicrobial resistance [26]. Although strict classification into additional pneumonia subtypes was limited by overlapping clinical features and retrospective design, the cohort encompassed the three major ICU pulmonary infection categories: general pulmonary infection, severe pneumonia, and aspiration‐related pneumonia. This grouping ensures that the observed diagnostic performance of tNGS reflects real‐world ICU practice.

A notable finding was the frequent detection of multiple microbial categories in individual specimens. While these sequencing‐level codetections do not necessarily represent confirmed coinfections, they reflect the complex microbial milieu in critically ill patients and are influenced by prior antimicrobial exposure and ICU patient complexity. Using amplification‐based tNGS, a broad pathogen spectrum encompassing bacteria, viruses, and fungi was observed, with bacterial pathogens accounting for the largest proportion. Co‐occurrence network analysis demonstrated recurrent mixed microbial detection patterns, with several pathogens occupying central positions, consistent with the known epidemiology of severe pulmonary infections [19]. Stratified analyses (Supporting Table S1) showed consistently high tNGS positivity across all age groups, sexes, comorbidities, and pneumonia subtypes. Polymicrobial detection was more frequent in patients aged ≥ 80 years and in BALF specimens, whereas most comorbidities and ICU diagnostic categories had limited impact, providing clinically meaningful insights into infection patterns. Compared with CMTs, sequencing‐based approaches may provide a more comprehensive overview of the microbial landscape, particularly in culture‐negative or pretreated patients, which is a common scenario in the ICU [27].

When clinical diagnosis was used as the reference standard, amplification‐based tNGS demonstrated substantially higher sensitivity and overall diagnostic accuracy compared with CMTs, whereas conventional methods showed higher specificity. This pattern aligns [19, 28, 29]. Lower specificity observed when benchmarking tNGS against a clinical reference standard should be interpreted cautiously [30], as respiratory specimens may harbor a range of microorganisms, including colonizing bacteria and latent viruses, and sequencing‐based approaches may detect nucleic acids from nonviable organisms or background microbial signals that are not causally related to disease. In respiratory infections, distinguishing infection from colonization or contamination is a recognized interpretive challenge, and different specimen types and analytical methods require distinct interpretive frameworks [30–32].

The higher tNGS positivity rate is clinically plausible in critically ill patients, where prior antimicrobial exposure and impaired host defenses are common [33]. However, this increased detection sensitivity also introduces a substantial interpretive burden. Clinicians must integrate sequencing results with clinical presentation, imaging findings, inflammatory markers, and conventional microbiological data to determine the clinical relevance of each detected organism [34]. Consequently, structured interpretive frameworks, contamination monitoring, and transparent reporting criteria are essential to ensure that high‐sensitivity molecular results support, rather than complicate, antimicrobial decision‐making and stewardship [35].

Several limitations of this study should be acknowledged. First, its single‐center, retrospective design may limit generalizability. Second, comorbidities were restricted to documented diagnoses in electronic medical records; undiagnosed chronic pulmonary conditions and unrecorded ENT disorders, sinusitis, or primary immunodeficiencies (other than HIV) may have been present. Third, the interval from symptom onset to specimen collection could not be reliably determined for all patients due to incomplete documentation, though specimens were typically collected within 72 h of ICU admission. Fourth, amplification‐based tNGS relies on predefined primer panels and therefore cannot detect pathogens outside the targeted range. Fifth, the co‐occurrence network analysis was based on detection frequency and cannot establish causal or synergistic relationships between pathogens. Finally, clinical diagnosis was used as the reference standard, which may introduce classification bias.

Despite these limitations, the study provides real‐world evidence regarding pathogen spectra, mixed microbial detection patterns, and the diagnostic performance of amplification‐based tNGS in critically ill patients. Amplification‐based tNGS offers a sensitive and comprehensive approach for pathogen detection. The frequent observation of mixed microbial detections highlights the complexity of ICU respiratory infections and underscores the importance of cautious interpretation of sequencing results. When integrated with clinical judgment and conventional microbiological testing, tNGS may serve as a valuable adjunctive tool to support etiologic diagnosis and informed antimicrobial management in the ICU setting.

## Author Contributions

Lin Yang: conceptualization, methodology, investigation, data curation, writing–original draft, and visualization. Zhao Zhao: methodology, investigation, data curation, writing–original draft, and formal analysis. Li Zhao: methodology and investigation. Shujuan Zhang: Methodology and investigation. Xiaozhi Jiang: software, investigation, and visualization. Fengdan Liu: validation and investigation. Jin Wu: Validation, investigation, and writing–review and editing. Lili Du: investigation, data curation, formal analysis, and writing–review and editing. Guannan Ma: conceptualization, software, and writing–review and editing. Peili Chen: conceptualization, resources, writing–review and editing, and funding acquisition.

## Funding

This work was supported by the Key Science and Technology Research and Development Program of Shangqiu (grant no. HS2025092).

## Ethics Statement

This study was approved by the Ethics Committee of Shangqiu First People’s Hospital (HS2025092). Given the retrospective nature of the study, the requirement for informed consent was waived.

## Conflicts of Interest

The authors declare no conflicts of interest.

## Supporting Information

Additional supporting information can be found online in the Supporting Information section.

## Supporting information


**Supporting Information** Supporting Materials. Table S1. Baseline clinical characteristics of critically ill patients with suspected pulmonary infection included in the study.

## Data Availability

The datasets analyzed during the study would be acquired from the corresponding author upon reasonable request.
